# Artificial Intelligence Chatbots in Surgical Care: A Systematic Review of Clinical Applications

**DOI:** 10.55578/joaims.250603.001

**Published:** 2025-07-22

**Authors:** Anne E. Hall, Amanda T. Perrotta, Kaavian Shariati, Archi K. Patel, Justine C. Lee

**Affiliations:** 1 Division of Plastic and Reconstructive Surgery, University of California Los Angeles, David Geffen School of Medicine, Los Angeles, CA, USA; 2 UCLA Gender Health Program, University of California Los Angeles, David Geffen School of Medicine, Los Angeles, CA, USA

**Keywords:** Chatbot, Conversational agents, Artificial intelligence, Surgery, Perioperative, Intervention, Application

## Abstract

**Background:**

In the context of surgical care where accurate and timely information is essential, artificial intelligence (AI)-driven chatbots offer innovative opportunities for improving patient education and perioperative outcomes.

**Methods:**

A systematic review per PRISMA guidelines was conducted to evaluate the application of chatbots within the surgical pathway and assess outcomes relating to patient experience, cost, safety, and clinical recovery. Studies were retrieved from MEDLINE, EMBASE, CENTRAL, and Google Scholar databases (November 2024) and were included if they deployed chatbots in the perioperative timeframe for adult surgical patients.

**Results:**

The review encompasses twelve studies totaling 6,619 patients, featuring rule-based, rule-and-frame-based, hybrid, and generative AI chatbots. Chatbots were used for delivering automated information (66%), answering patient queries (66%), symptom monitoring (16%), facilitating clinic communication (16%), and soliciting patient feedback (8%). Chatbots achieved 60–82% satisfaction on Likert scales, engagement rates of 35–83%, and accuracy rates from 79–99% depending on function. Additionally, they reduced healthcare personnel workload, saving 9–39 hours per 100 patients postoperatively. While no shared clinical outcomes were assessed across multiple studies, individual studies found chatbot use was associated with reductions in opioid use, pain, preoperative anxiety and readmissions. Risk of bias was assessed via the ROBINS-I tool with most studies classified as low risk (67%) and 25% as serious risk due to confounding variables, missing data, and measurement biases.

**Conclusion:**

Chatbots are emerging as a useful tool in surgical care, improving patient outcomes, satisfaction, and engagement. Future research can further explore chatbot models, delivery methods, and surgery-specific applications on clinical outcomes.

## 1. INTRODUCTION

Artificial intelligence (AI) has evolved over time, transitioning from rudimentary rule-based support systems, which rely on predefined “if-then” logic to make decisions, to frame-based systems, which structure knowledge into conceptual hierarchies for context-aware reasoning [1,2]. These approaches, while effective for specific tasks, were limited in flexibility and scalability. The development of hybrid systems, which combine symbolic reasoning (rule- and frame-based logic) with data-driven techniques, marked a step toward more adaptive AI, where sophisticated frameworks such as natural language processing (NLP) and machine learning (ML) were created to enable AI to analyze and learn from unstructured data [3]. More recently, generative AI has emerged, capable of extrapolating beyond existing data and patterns [4]. As AI advances in this manner, its implementation continues to expand across diverse disciplines, serving increasingly consequential roles. With the ongoing innovation in healthcare technology, AI is gaining attention for its implementation in diagnostics, administrative workflow automation, and medical education [5–8].

In addition to being utilized by providers, AI is becoming increasingly relevant in direct patient care. Among its applications, chatbots—AI-powered conversational agents—have emerged as one of the most practical, accessible and scalable tools [9]. These systems, designed to simulate human interaction, offer an innovative means to provide personalized, timely, and accurate support to patients. The majority of prior data pertains to mental health applications, where clinical chatbots have been used to deliver therapeutic interventions, conduct diagnostic screening, assist with self-monitoring and behavior prompting, and facilitate cognitive restructuring, highlighting their potential to promote meaningful patient engagement and personalized support [10–12]. Beyond mental health, conversational agents have improved self-management and treatment adherence across various conditions such as asthma, HIV, and breast cancer, while also promoting healthier lifestyle behaviours [13–16]. Recently, Laymouna et al. conducted a systematic review of 161 studies wherein chatbots were used to deliver remote health services including patient support, care management, education, skill building, and health behavior promotion [17]. 53.7% of the included studies reported improved health outcomes and patient management, while 47.8% highlighted reductions in administrative or financial burdens on the healthcare system [17]. Elsewhere, other systematic reviews have emphasized high levels of patient satisfaction, usability, and engagement, with abundant opportunities for personalization across healthcare chatbot interventions [18,19].

In the context of surgery, chatbots offer a means to provide patient education and surgical information, while addressing inquiries, concerns, and questions in a timely manner. For instance, chatbots can provide postoperative recovery guidance, increase awareness of potential complications, and assist with symptom monitoring [20]. While less is certain regarding the role of chatbot integration in the context of surgery relative to other healthcare fields, the limited prior data available shows promise. A systematic review by Lin et al. found that majority of participants were satisfied with the use of chatbots (mean proportion = 0.73) and gained knowledge in the perioperative period (mean proportion = 0.80) [21]. However, this study was constrained by a small number of studies and participants, and it classified many chatbots solely as rule-based, highlighting limitations in the complexity and adaptability of current chatbot and AI systems.

This review seeks to evaluate chatbot interventions designed to deliver surgery-related communication and analyze their impact on patient experiences, cost-effectiveness, safety, and clinical outcomes. By identifying trends, challenges, and opportunities in perioperative chatbot implementation, this review aims to offer insights on the evolving intersection of AI and surgical care.

## 2. METHODS

### 2.1. Search Criteria

A systematic literature review was performed following the Preferred Reporting of Systematic Reviews and Meta-Analysis (PRISMA) guidelines [22]. A comprehensive search of MEDLINE, EMBASE, CENTRAL, and Google Scholar databases was conducted in November 2024, utilizing terms such as “chatbot”, “conversational agent”, “surgery”, “perioperative”, “surgical patients”, “intervention” “implementation”, “applied”, and “outcome” (Supplementary Figure 1). Only articles published in English were included.

### 2.2. Inclusion and Exclusion Criteria

The initial screening of all identified abstracts were performed by two independent reviewers (AEH and ATP). Inclusion criteria for full-text review encompassed studies involving adult patients undergoing surgical procedures, the use of chatbots in the perioperative setting, and the reporting of relevant outcomes such as opioid use, pain, quality of life, patient experience, chatbot performance metrics or cost-effectiveness with validated measures or scoring systems. All studies that met this criteria were considered for full-text review.

A chatbot intervention was defined as any automated system designed to interact with patients via text or voice, providing information, assistance, or follow-up assessments within the context of perioperative care. No limitations were placed regarding the type of chatbot employed. Chatbots were categorized as rule-based, frame-based, hybrid, or generative AI. Rule-based chatbots operate without AI components, relying solely on predefined rules and templates, offering rigid responses. Frame-based chatbots combine structured workflows and slot-filling mechanisms to guide conversations within predefined parameters. Hybrid models integrate structured rule-based frameworks with more advanced AI techniques like NLP and ML to interpret input and provide context-specific responses within defined parameters. Lastly, generative AI chatbots, leverage large language models (LLMs), such as ChatGPT and Google Gemini, and deep learning to process unstructured input and generate dynamic, human-like responses without relying on predefined rule-based frameworks [23,24].

Studies were excluded if the chatbot was utilized during the consent process, for diagnostic purposes, or as a decision-making tool. Additionally, studies were excluded if the primary function of the chatbot was limited to remote patient symptom monitoring without bidirectional interaction (i.e., the ability for patients to actively engage and initiate communication with the chatbot). These criteria ensured that the review focused specifically on chatbots designed to facilitate patient communication and interaction throughout the surgical care pathway.

The reference lists of all included full-text articles, as well as other relevant articles identified during the screening process, were examined to locate additional studies.

### 2.3. Assessment of Study Quality

The risk of bias for quantitative outcomes was assessed using the revised version of the ROBINS-I tool (Risk of Bias in Non-randomized Studies-of Interventions) by two independent reviewers (AEH and ATP) [25]. The ROBINS-1 tool evaluates bias across seven domains: bias due to confounding variables, selection of participants into the study, classification of interventions, deviations from intended interventions, missing data, measurement of outcomes, and selection of the reported result. Each outcome measure within a study was rated as having a low, moderate, serious, or critical risk of bias, and these ratings were used to determine an overall risk of bias score for each study. Discrepancies between reviewers were resolved through discussion.

### 2.4. Data Extraction

Study characteristics were independently extracted by AEH and ATP, including study type, sample size, and surgical subspecialty. Chatbot intervention characteristics included the phase of care during which the chatbot was deployed, the type of chatbot, the delivery method, and a brief description of the chatbot. The follow-up duration of the study as well as relevant outcome measures such as opioid use, pain, quality of life, patient experience and engagement, and cost-related and safety outcomes and the type of measure utilized were also collected. Any data not reported in the original studies are labeled as “not available” (NA) in the corresponding table.

### 2.5. Statistical Analysis

Descriptive statistics were used for analysis. All cost and time-related calculations were derived from data tables provided in the studies. For studies that did not explicitly calculate time savings, the total hours reduced per 100 patients was estimated by analyzing standard care and chatbot implementation costs. A detailed outline of the calculations is provided in Supplementary Figure 2.

## 3. RESULTS

### 3.1. Study Characteristics

**Figure 1 |:**
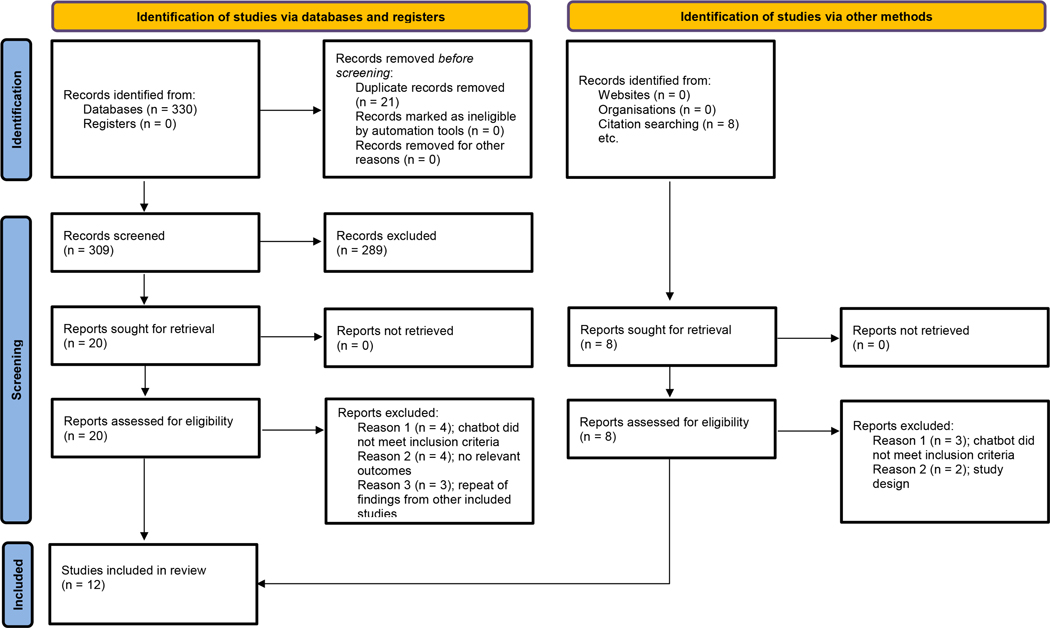
PRISMA flowchart. PRISMA refers to preferred reporting items for systematic reviews and meta-analyses.

A search of MEDLINE, EMBASE, CENTRAL, and Google Scholar databases resulted in 339 articles for applied chatbot interventions (Figure 1). After abstract and full-text review screening, 12 articles were included (Table 1) in our applied chatbot interventions review [26–37].

The included studies, published between 2019 and 2024, encompass 6,619 patients undergoing various surgical procedures (intervention group, n = 3,593; control group, n = 3,026; Table 2). Among the various study designs, the most common were were prospective cohort studies and feasibility studies (33.4%), followed by prospective observational or comparative studies (25.0%), randomized controlled trials (16.7%), retrospective reviews (16.7%), and randomized trials (8.3%). Six studies included a control group in which patients received standard perioperative care with no chatbot.

Most studies focused on patients undergoing orthopedic surgeries, accounting for 6 out of the 12 included studies (50.0%). The types of procedures within orthopedic surgery were variable, but included total joint arthroplasty (hip and knee), hip arthroscopy, total knee replacement, and trauma surgeries. Other specialities included anesthesia (16.7%), colorectal surgery (8.3%), ophthalmology (8.3%), urology (8.3%), and vascular surgery (8.3%). Chatbots were most commonly deployed in the postoperative phase of care (66.7%), followed by both the preoperative and postoperative phases (16.7%), and the preoperative phase only (16.7%).

Regarding chatbot type, seven studies employed hybrid chatbots, two employed rule-and-frame based chatbots, two used solely rule-based chatbots, and one utilized a generative AI chatbot (ChatGPT 3.5). Chatbots were primarily accessed via smartphones (83.3%), with delivery methods including text (60.0%), mobile apps (20.0%), and phone calls (20.0%). One study employed a website URL that could be accessed from any smart device (e.g., smartphone, tablet, or computer) [31] and another had patients utilize ChatGPT-3.5 [37].

In terms of chatbot use, 41.7% of studies utilized chatbots to specifically facilitate postoperative care providing educational resources, recovery instructions, symptom management, rehabilitation guidance, complication monitoring, and real-time support, including symptom tracking and clinical team referrals. An additional 33.3% developed chatbots to assist with overall surgery support. This included providing personalized preoperative preparation tips, recovery guidance, therapy videos, FAQs, answers to patient concerns (e.g., pain management, showering protocols), and personalized messages from surgeons to enhance patient care and engagement both before and after surgery. 16.7% utilized chatbots via automated postoperative follow-up calls, collecting feedback on hospital experiences, recovery, and rehabilitation, or conducting symptom assessments with real-time supervision. Lastly, 8.3% specifically used the chatbot to help deliver acceptance and commitment therapy to patients postoperatively. Overall, regarding chatbot function, 66.67% of chatbots provided information to patients, 66.67% answered patient queries, 16.67% assisted with symptom monitoring and complication detection, 16.67% facilitated follow-up appointments and clinic communication and 8.3% solicited patient feedback.

### 3.2. Applied Chatbot Integration Outcomes

**Figure 2 |:**
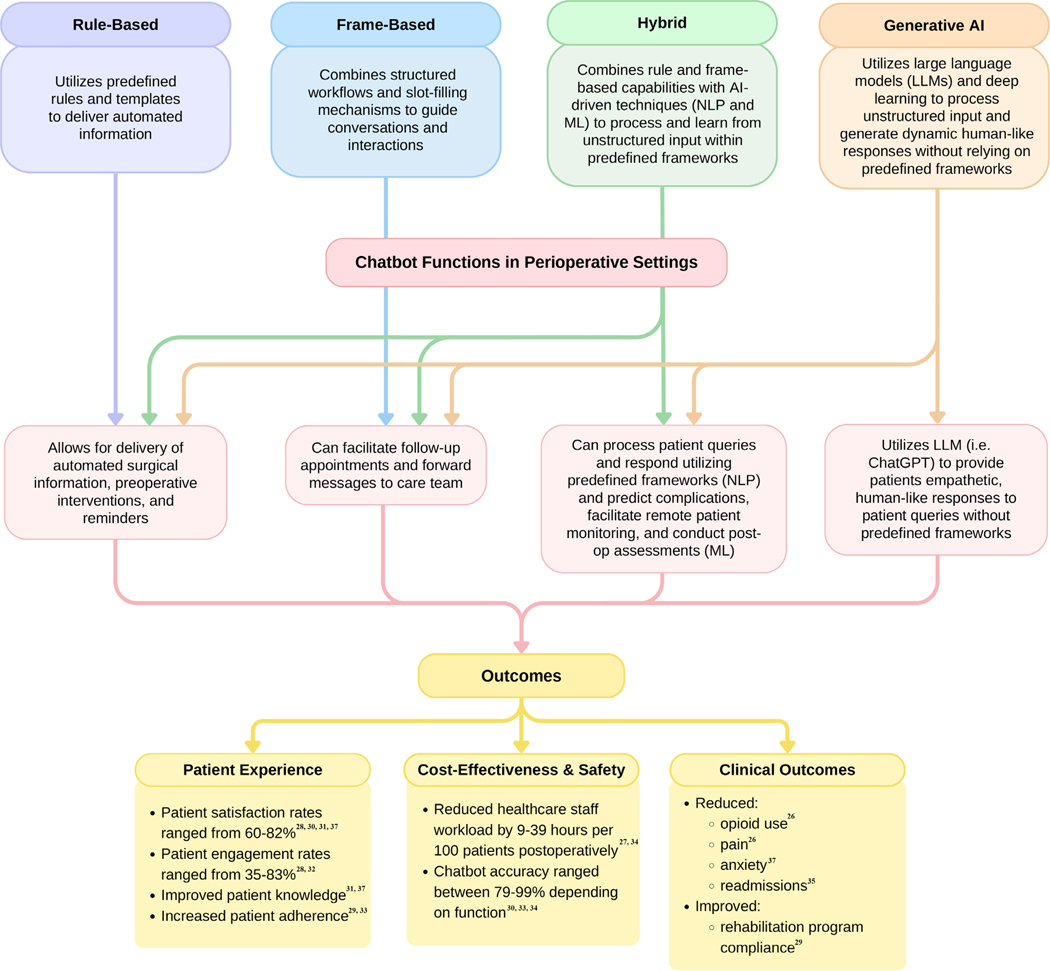
Chatbot flowchart. This figure summarizes the different chatbot types, associated functions in the perioperative setting, and outcomes reported in this review.

Studies examined a broad range of outcomes, which were grouped into three key categories: patient experience, cost-effectiveness and safety, and clinical outcomes (Figure 2).

Eleven studies reported on various outcomes related to patient interaction with chatbots, with five studies examining patient satisfaction [28,30,31,33,37], three studies on perceptions [32,34,37], four studies on engagement [28,32,35,36], two addressing knowledge or understanding [31,37], and two addressing patient adherence [29,33]. Overall, high satisfaction rates were reported, with 60.1–82.0% of patients reporting the chatbot was helpful or delivered clear and appropriate information (measured through Likert scales) [28,30,31,37]. While patients generally viewed chatbots positively, especially regarding with the chatbot’s utility for non-urgent concerns, one study raised comments from patients about the lack of a “human element,” in complications, while another noted a preference for calling the clinic for serious issues [32,34].

Engagement rates ranged from 35.0–83.3%, with one study identifying primary barriers to engagement to be misplacing follow up instructions or relying on follow–up with clinic or discharge papers [28,32]. In the studies evaluating language, chatbot engagement was found to be comparable between patients with limited English proficiency and English as primary language [35]. Additionally, a study comprising 1,338 patients identified factors associated with higher engagement, including female sex, non-Medicaid insurance, older age, better pre- and postoperative physical function, and lower preoperative pain scores [36]. Conversely, lower engagement was observed in patients with higher readmission rates [36].

Of studies evaluating patient knowledge or understanding, one study assessed patient knowledge through a self-reported anesthesia knowledge test before and after the chatbot was delivered, finding a significant improvement in knowledge following chatbot use compared to control group (p < 0.001) [31]. The other study evaluated improvement of patients’ understanding of their treatment process after a chatbot was used for preoperative consultation, reporting an average improvement score of 2 out of 4 [37]. Of studies assessing patient adherence among the chatbot intervention group, one reported a 78.0% adherence rate to a rehabilitation program (attending > 80.0% of program sessions) [29], while the other found 83.7% complete adherence to daily postoperative symptom monitoring [33].

Four studies explored system-level outcomes, including cost-effectiveness [27,34], health resource utilization [30], and chatbot safety outcomes [30,33,34]. In studies that utilized chatbots in conducting patient feedback and follow-up assessments, it was found that chatbots were effective in reducing between 9.3–39.0 hours spent per 100 patients and cost benefits were £35.18 per patient with 96.5% of calls completed autonomously (Supplementary Figure 2) [27,34]. While one study did not conduct a cost-analysis, it examined health resource utilization, reporting that 46.0% of patients who were concerned about potential complications sought reassurance through the chatbot rather than pursuing additional medical attention. The chatbot responses to the patient queries were reviewed and found to be appropriate [30].

Three studies assessed chatbot safety-related outcomes, focusing on the accuracy of chatbot responses and its ability to identify and manage risks. Chatbot accuracy varied depending on function, ranging from 79.0% to 98.7% [30,33,34]. In one study evaluating patient inquiries, it was reported that the chatbot appropriately handled 79.0% of questions, correctly identifying topics 58.0% of the time, and correctly answering 31.0% of the cases independently [30]. Among questions having safety implications, the chatbot correctly responded to 70.0% of questions, though 30.0% were inadequately addressed, none resulted in patient harm.

In detecting complications, key symptoms, and determining follow-up needs, chatbot performance was mixed. Sensitivity ranged from 75.0% to 93.8% and specificity from 83.0% to 99.5% with error rates as low as 1.3% for symptom tracking but reaching 17.7% for complication detection [33,34]. Chatbots performed best in recognizing individual symptoms (98.5% accuracy) but had lower accuracy (88.7%), when assessing whether patients required further clinical management [34]. Complication detection was less reliable, with an accuracy of 82.0%, sensitivity of 75.0%, and a false negative rate of 25.0% [33]. Across studies, chatbots were more likely to produce false positives than false negatives, with false-positive rates ranging from 13.7–17% and false-negative rates from 6.3–25.0%, indicating that chatbots tend to overflag rather than miss concerns [33,34]. Importantly, even when chatbots failed to identify complications or flagged unnecessary concerns, no reported errors resulted in patient harm.

In clinical decision-making, one study found that chatbot recommendations matched physician discharge and review decisions 89.0% of the time [34]. While it correctly identified 58.0% of discharges and 31.0% of reviews, it tended to be overly cautious, generating more false positives (9.0%) than false negatives (2.0%). Overall, the chatbot demonstrated moderate-to-strong agreement with clinicians, reinforcing its reliability in postoperative management (k = 0.758–0.970).

Four studies assessed key measures of pre- and postoperative recovery, each evaluating different clinical outcomes. While no single outcome was measured across multiple studies, individually assessed outcomes included opioid use [26], pain intensity [26], anxiety [37], emergency department (ED) visits [35], readmissions [35], reoperation rates [35], and rehabilitation program adherence [29]. Validated measures were used in 80.0% of studies reporting patient-reported outcomes. Overall, chatbots demonstrated reduced opioid use (–37.0%) [26], pain (–3.7 points) [26], preoperative anxiety [37], and readmissions [35]. Additionally, one study reported a 15.0% increase in compliance with a postoperative rehabilitation program compared to the control group [29].

### 3.3. Bias Results

ROBINS-1 was used to assess the quality of included studies, with results summarized in Supplementary Figure 3. Of the 12 studies meeting inclusion, 8 were classified as low risk (66.7%), 1 of moderate risk (8.3%), and 3 of serious risk (25.0%) of bias. The quality of the included studies was assessed for each quantitative outcome measure individually. However, as there were no differences in the risk assessments across individual outcome measures, the overall quality ratings are reported collectively.

Downgrades in the risk of bias score were due to confounding variables where studies did not adequately control for external variables [28,32], deviations from intended interventions [31], missing data [28], and measurement of outcomes [26]. For example, Black et al. and Goldenthal et al. did not control or evaluate confounding variables in their patient populations, introducing unaccounted external factors. Ferre et al., conducted a per-protocol analysis, including only patients who accessed the intervention website, introducing the potential for selection bias. Black et al., also lacked clear information about how data on engagement and satisfaction were collected, raising potential concerns about missing data and response validity. Anthony et al., demonstrated possible measurement bias, as research assistants and patients were not blinded to patient group assignments or outcomes of interest.

Among studies that were considered low risk and serious risk, 18.2% consisted of abstracts, limiting the ability to assess the full methodologies, evaluate the robustness of outcome measures, and determine the extent of potential bias.

## 4. DISCUSSION

The capabilities and integrations of AI in healthcare are increasing rapidly, offering the potential to enhance patient and clinician experiences and overall healthcare efficiency [5,38,39]. The current study investigated the role of chatbot applications within the surgical care pathway and found that they offer utility across all stages of perioperative care, particularly in education, personalized support, symptom monitoring, follow-up assessments and clinic communication. Patient satisfaction was consistently high (60.1–82.0%), though engagement rates varied (37.0–83.2%). Chatbots demonstrated high accuracy in symptom identification (98.5%), but were less so in responding appropriately to patient queries (79.0%). They also contributed to a reduction in healthcare personnel workload, saving 9.3–39.0 hours per 100 patients. While evidence on clinical outcomes remains limited to individual studies, chatbots were associated with reduced opioid use, pain, preoperative anxiety, and readmissions.

Across the examples of chatbots integrated into the surgical setting thus far, this review identified a predominance of hybrid chatbots. Hybrid frameworks integrate structured dialogue systems for interaction management with NLP for both enhanced language understanding and ML for predictive analysis and learning capabilities. This combination enables the processing of unstructured data, while maintaining predictability, adaptability, and flexibility, suggesting the suitability of hybrid chatbots in addressing the specific, dynamic needs of surgical support [40]. These systems utilize rule-based logic to deliver surgery- and surgeon-specific information, while leveraging AI-driven adaptability to personalize patient interactions with responses informed by prior conversations, sentiment analysis, and real-time user input. This enables the delivery of precise, procedure-specific instructions while also recognizing variations in patient variations in patient inquiries, allowing for more natural and interactive engagement [41]. Some approaches, such as that employed by Rainey et al. are further augmented by therapy videos and personalized video messages from providers, reflecting opportunities for customization beyond surgical information and integration of multimedia components to improve patient comprehension and engagement [35,36].

Additionally, many of the chatbots included in this review performed several functions simultaneously, highlighting their multifaceted perioperative roles. Beyond delivering surgical information and answering patient queries in real time, hybrid chatbots also solicited feedback through automated calls, facilitated follow-up appointments, and improved overall clinic communication. Additionally, their ML-driven predictive capabilities allowed them to perform relevant functions independently of conversations directly initiated by patients to monitor patient symptoms, conduct follow-up assessments, and predict early complications. These advanced AI-driven capabilities differ from the more elementary rule-based systems and frame-based systems, which can only deliver pre-programmed responses based on fixed decision trees, lacking the ability to independently assess patient status and interpret unstructured user inquiries.

However, while hybrid chatbots enhance adaptability compared to purely rule- and frame-based models, they still operate within predefined structures and learning parameters, which may limit their ability to handle complex, nuanced, or unforeseen patient inquiries. This presents an opportunity to integrate more advanced AI models, such as generative AI, to enhance chatbot functionality and responsiveness. Unlike hybrid systems, generative AI-driven chatbots may allow for more fluid, adaptive, and personalized interactions beyond predefined frameworks, informed by much broader knowledge and evidence bases [42]. As such, generative AI models may enable chatbots to better understand open-ended patient concerns and provide more refined, natural, and context-responses mirroring the communication style of healthcare providers [43,44]. Future research may prioritize the integration of generative AI into existing chatbot frameworks to improve patient-centered, personalized interactions. This could include adapting content based on individual patient demographics, surgical procedures, health literacy levels, and emotional tone. By leveraging generative models, chatbots can provide tailored education, answer context-specific questions, and reference surgeon- or procedure-specific information in real time, ultimately strengthening relevance and engagement throughout the perioperative process. However, barriers relating to the accuracy, safety, validity and reliability of LLMs, such as ChatGPT and Google Gemini, warrants further investigation as generative AI is also prone to bias and “hallucinations,” where LLMS may fabricate information or cite non-existent sources [43].

Beyond refining chatbot models, future efforts may seek opportunities to expand the deployment of chatbots across the entire perioperative timeline, with tailored functionalities at different timepoints. In this review, the majority of chatbots were deployed in the postoperative phase, with only 16.7% of studies integrating them both pre-and postoperatively [35,36]. In these applications, chatbots provided information pre- and postoperatively and allowed patients to interact with the chatbot throughout for support. This demonstrates that employment throughout the entire perioperative pathway may further optimize comprehensive surgical care, providing preoperative education, intraoperative updates, and postoperative recovery support. Additionally, to maximize their impact and accessibility, further research may explore the integration of chatbots into mobile health (mHealth) applications. While only two studies in this review delivered the chatbot within a mobile app, they reported high engagement, compliance, retention, and satisfaction, presenting an opportunity to consolidate chatbot functionalities into a single, accessible digital health platform [28,29]. The integration of chatbots within mHealth apps have been piloted as seen with the BalanceUp App, a smartphone-based coaching intervention aimed to improve the mental well-being of individuals experiencing frequent headaches [45]. The app features a chat-based interface for communicating with the conversational agent, audio-guided relaxation exercises, animated psychoeducational videos, informative schematics, and frequently asked questions, and was found to improved well-being, and perceived stress. In the context of perioperative support, a mobile app could serve as a centralized hub, not only delivering chatbot-driven support, but also presenting surgery-specific information in a multimedia format, integrating patient engagement features, and incorporating health data such as physical activity tracking, sleep monitoring, nutritional guidance and stress management.

While chatbots offer promising applications in the surgical pathway, several challenges must be addressed to facilitate their successful development, implementation and widespread adoption. Designing and deploying a customizable chatbot with multiple functionalities requires significant resources with multidisciplinary effort from developers, clinicians and healthcare institutions. Additionally, ensuring that chatbots effectively meet patient needs necessitates thorough patient and community needs assessments, which can be complex and resource-intensive, but necessary to improve usability and foster patient buy-in and sustained engagement. However, achieving high patient engagement can be challenging, particularly if patients perceive chatbots as impersonable, lacking reliability, and conveying information at an advanced reading level. As concerns remain for safety, clinical oversight, and reliability of guidance, establishing clear protocols for accuracy, clinical validity and data security is essential to effectively detect complications and avoid misinformation. In particular, the protection of patient privacy and personal health information would benefit from being prioritized, especially as chatbot technologies increasingly interface with electronic health records and other sensitive data systems. Addressing these challenges through rigorous evaluation, continuous refinement and strategic integration into existing healthcare frameworks will be essential for maximizing the impact of chatbots in chatbots in surgical care.

This systematic review is subject to several limitations. First, the number of included studies was constrained by the limited research on perioperative chatbot deployment, reflecting the early-stage exploration of this technology in surgical care. Several of the included studies were feasibility trials or conference abstracts, demonstrating the novelty of perioperative chatbots and the need for more robust, large-scale investigations. Outcome measures across were also heterogeneous, ranging from patient-reported clinical outcomes to chatbot performance metrics and operational feasibility. This variability limited the ability to synthesize findings and conduct a meta-analysis, reducing the generalizability of conclusions. The lack of standardized clinical endpoints also makes it difficult to compare intervention effectiveness across contexts or to draw definitive conclusions about chatbot impact on surgical outcomes. Future research can prioritize standardized outcome reporting to enhance comparability across studies and include extended follow-up periods to assess the long-term effectiveness and sustainability of chatbot interventions. Establishing consistent clinical and usability metrics will facilitate more rigorous reviews, ultimately guiding the development and optimization of chatbot interventions in surgical care. Lastly, while a quarter of the included studies were rated as concerning for serious risk of bias, potentially limiting the strength of our overall conclusions, key outcomes related to satisfaction, cost-effectiveness, and accuracy were generally consistent across studies, regardless of bias level. Nevertheless, future investigations employing more rigorous methodologies are needed to validate and expand upon these findings.

## 5. CONCLUSION

The systematic review performed within this study demonstrated that chatbots are being widely implemented in the perioperative context largely providing surgical information, symptom management and tracking, rehabilitation guidance, complication monitoring, and follow-up assessments. Overall, our findings demonstrate high patient satisfaction and engagement, while improving cost-effectiveness and safety in perioperative care. They also have the potential to reduce healthcare personnel workload and improve opioid use, pain, anxiety, and readmissions. While further research is needed to validate these findings across larger studies, the integration of chatbots whether—rule-based, frame-based, hybrid, or generative AI—represents a promising avenue for improving surgical outcomes, postoperative monitoring, and the patient experience.

## Supplementary Material

1

## Figures and Tables

**Table 1 |:** Study and chatbot intervention characteristics

References	Study type	Sample size (I:C)	Surgical subspecialty (Procedure)	Phase of care	Chatbot type	Delivery method	Follow-up period	Chatbot description

Anthony et al., 2020 [26]	RCT	76 (38:38)	Orthopedic surgery (trauma fracture fixation)	Post-op	Rule-based	Smartphone (text)	2 weeks	**ACT Therapy:** Chatbot delivered ACT messages twice daily to help patients manage post-op pain, reduce opioid usage, and improve emotional well-being.
Bian et al., 2020 [27]	Prospective comparative study	2926 (270:2656)	Orthopedic surgery (various)	Post-op	Hybrid	Smartphone (call)	1–2 months	**Solicited Patient Feedback:** Chatbot delivered automated follow-up calls using a human-like voice. It gathered patient feedback on hospital stay, health education, wound healing, rehabilitation, complications, and other surgery-related concerns. With advanced speech recognition, the agent detected different patient dialects and converted voice input to text in real time.
Black et al., 2020 [28]	Prospective observational study	168 (168:0)	Vascular surgery (MIS venous interventions)	Post-op	Rule-based and frame-based	Smartphone (app)	1 week, 1 month, 3 months	**Post-Op Support:** Chatbot delivered automated care by reinforcing post-op instructions regarding activity, wound care, compression, and awareness of potential complications, while also facilitating follow-up appointments and clinic communication.
Blasco et al., 2022 [29]	Randomized trial	18 (9:9)	Orthopedic surgery (TKR)	Post-op	Hybrid	Smartphone (app)	3 months	**Post-Op Support:** NLP chatbot delivered automated reminders, support, motivational messages, and multimedia education for rehabilitation exercises.
Dwyer et al., 2023 [30]	Prospective cohort study	26 (26:0)	Orthopedic surgery (hip arthroscopy)	Post-op	Hybrid	Smartphone (text)	6 weeks	**Post-Op Support:** Chatbot delivered automated messages regarding pain management, wound care, physiotherapy, and rehabilitation, and performed wellness checks, responded to patient questions, and forwarded concerns to the care team as needed.
Ferré et al., 2020 [31]	Prospective observational trial	303 (98:205)	Anesthesia	Pre-op	Rule-based	Smart device (website)	Immediately after PAC	**Surgery Support:** Chatbot delivered knowledge about the perioperative care pathway featuring themed sections (i.e. team, support, technique, and recovery room) each including videos, pictures, and FAQs with corresponding answers. A synthesized voice read the written information, and videos were subtitled to optimize patients’ comprehension.
Goldenthal et al., 2019 [32]	Feasibility study	20 (20:0)	Urology (ureteroscopy)	Post-op	Rule-based and frame-based	Smartphone (text)	1–4 weeks	**Post-Op Support:** Chatbot provided support to address common post-procedure symptoms and patient concerns, while providing educational information.
Kupper et al., 2024 [33]	Prospective cohort study	92 (92:0)	Colorectal surgery (colorectal cancer surgery)	Post-op	Hybrid	Smartphone (text/email)	15 days post-discharge	**Post-Op Support:** Chatbot was integrated within a digital monitoring platform to track patient symptoms and predict early complications. Chatbot initiated dialogue and answered patient questions and concerns during recovery.
Meinert et al., 2024 [34]	Feasibility study	202 (202:0)	Ophthalmology (cataract surgery)	Post-op	Hybrid	Smartphone (call)	3 weeks	**Post-Op Assessments:** Chatbot conducted follow-up symptom assessments and answered patient questions via telephone, with real-time supervision by two ophthalmologists.
Rainey et al., 2023 [35]	Retrospective review	1,350 (1282:68)	Orthopedic surgery (TKA, THA)	Pre-op, post-op	Hybrid	Smartphone (text)	90 days	**Surgery Support:** Chatbot delivered automated multilingual SMS text messages with surgery preparation, recovery information, therapy videos, and personalized video messages from their surgeon. Patients could also initiate conversations with the chatbot and inquire about various topics relating to surgical care.
Rainey et al., 2024 [36]	Retrospective review	1,338 (1338:0)	Orthopedic surgery (TKA, THA)	Pre-op, post-op	Hybrid	Smartphone (text)	90 days	**Surgery Support:** Chatbot delivered automated messages to patients including preoperative preparation tips, post-op recovery, therapy videos, and personalized video messages directly from their surgeons. Patients could also initiate conversations with the chatbot and inquire about various topics relating to surgical care.
Yahagi et al., 2024 [37]	RCT	100 (50:50)	Anesthesia	Pre-op	Generative AI	ChatGPT 3.5	1 hour after consultation and 1 day after surgery	**Surgery Support:** Patients were encouraged to interact with ChatGPT to seek information or address concerns related to surgery or anesthesia. They were assured that the medical information provided by ChatGPT would be reviewed by the anesthesiologist to identify any discrepancies and ensure patient safety and accuracy.

ACT: Acceptance and Commitment Therapy; AI: Artificial Intelligence; C: Control Group; ED: Emergency Department; EPL: English Proficient Language; FAQs: Frequently Asked Questions; I: Intervention Group; LEP: Limited English Proficiency; MIS: Minimally Invasive Surgery; NLP: Natural Language Processing; PAC: Preanesthetic Consultation; RCT: Randomized Controlled Trial; SMS: Short Message Service; THA: Total Hip Arthroplasty; TKA: Total Knee Arthroplasty; TKR: Total Knee Replacement.

**Table 2 |:** Summary of findings

Study	Category	Outcome	Metric	Finding

Anthony et al., 2020 [26]	Clinical outcomes	Opioid use	Patient opioid tablets	The IG used 36.5% less opioid tablets than the CG (p = 0.004).
	Clinical outcomes	Pain-intensity	PROMIS pain intensity 3A	The IG reported a lower post-op pain score than the CG (45.9 ± 7.2 vs 49.7 ± 8.8, p = 0.04).
	Clinical outcomes	Pain interference	PROMIS pain interference 8A	The IG reported a lower post-op pain interference score than the CG (56.6 ± 9.4 vs 60.6 ± 8.2, p = 0.05).
	Clinical outcomes	Emotional distress-anxiety	PROMIS emotional distress-anxiety 8A	There was no significant difference in post-op anxiety between the IG and CG (p = 0.76).
Bian et al., 2020 [27]	Cost and safety outcomes	Time spent / cost-effectiveness	Hours spent	Time spent on feedback collection per 100 patients was 9.3 hours in CG vs 0 hours in IG.
	Patient experience	Feedback collection rates	(Number of patients with effective feedback / number of effective follow-ups) X 100%	Better feedback collection rates with the conversational agent (IG) compared to manual follow-up (CG) (10.3% vs 2.5%, p < 0.001).
	Patient experience	Feedback composition	Quantitative content analysis	IG: 53.6% of feedback focused on hospital environment, followed by nursing (28.6%), medical consultation (10.7%), and health education (7.1%). CG: 87.0% of feedback focused on medical consultation, followed by nursing (7.3%), hospital environment (4.3%) and health education (1.4%).
Black et al., 2020 [28]	Patient experience	Patient engagement	NA	The chatbot demonstrated 83.3% engagement rate.
	Patient experience	Patient satisfaction	NA	60.1% of patients reported a high degree of satisfaction and found the chatbot to be helpful or very helpful in their care.
Blasco et al., 2022 [29]	Clinical outcomes	Rehabilitation program compliance	Percentage of patients that achieved adherence (>80.0% of rehabilitation sessions)	There was a 15.0% higher compliance of IG compared to CG. 78.0% of patients in IG achieved program adherence.
	Patient experience	Feasibility	Recruitment and retention rates	Patient recruitment was 75.0% with >85.0% retention to the chatbot intervention.
Dwyer et al., 2023 [30]	Patient experience	Patient satisfaction	5-point Likert scale	Average satisfaction of chatbot with post-op care: 4.0 ± 0.7. 80.0% of patients agreed or strongly agreed that the chatbot helped them understand what they need to know to manage their condition at home.
	Cost and safety outcomes	Chatbot accuracy	Appropriateness (Qualitative analysis of responses, categorization into ‘appropriate’ or ‘inappropriate’ handling and tracking performance metrics)	79.0% of patient questions were handled appropriately, with the chatbot either addressing questions independently or facilitating contact with the care team.
			Topic recognition	Chatbot correctly identified topic 58.0% of the time.
			Examples of confusion	Chatbot demonstrated confusion 63.0% of the time. Confusion significantly reduced over time during the study period (p = 0.03). Of the times chatbot was confused, chatbot could recognize its confusion in 65.0% of interactions.
	Cost and safety outcomes	Chatbot reliability	Percentage of questions adequately addressed by chatbot	Chatbot adequately addressed questions independently 31.0% of the time.
	Cost and safety outcomes	Safety	Questions were assessed by 2 reviewers and were identified as “safe,”“potentially unsafe,” or “unsafe” responses based on detailed criteria	10/128 (7.8%) of patient questions were identified as having safety implications, of which the chatbot correctly responded to 7 of them (70.0%) by providing correct advice or forwarding the question to the surgeon or instructing the patient to seek care. The 3 remaining responses were identified as ‘potentially unsafe’ from manual review, but none resulted in patient harm.
	Cost and safety outcomes	Health resource utilization	1. Total number of health care phone calls, emails, and visits related to surgery 2. Number of times the chatbot instructed the patient to seek care without directly forwarding the message to the surgeon.	13/26 patients combined for a total of 40 extra contacts with the healthcare system beyond the regular follow-up appointments over the first 6 post-op weeks. 12 patients were worried about a complication but were reassured by chatbot and did not seek medical attention (these instances were reviewed and found to be appropriate).
		Surgeon satisfaction	5-point Likert scale	Three surgeons rated satisfaction as excellent or good and all three agreed or strongly agreed they would be happy to continue to use the chatbot in other post-op scenarios.
Ferré et al., 2020 [31]	Patient experience	Patient knowledge	Self-reported anesthesia knowledge test	Patient knowledge about their perioperative care pathway improved significantly in IG compared to CG (1.7 vs. 1.0, p < 0.001).
	Patient experience	Patient satisfaction	5-point Likert scale for quality and accessibility	82.0% of patients found the chatbot delivered clear and appropriate information, and 74.0% found it easily accessible.
Goldenthal et al., 2019 [32]	Patient experience	Patient engagement	Number of patients that activated the chatbot	35.0% (7/20) patients activated the chatbot. Reasons for not activating chatbot included misplacing follow-up instructions (n = 6), relying on follow-up with the clinic or with discharge papers provided by the clinic (n = 4), being unable to access the chatbot (n = 2), and not using text messaging (n = 1).
	Patient experience	Patient perceptions	Semi-structured interviews	Patients found the chatbot was helpful for non-urgent concerns, offering quick information, though some preferred calling the clinic for more serious issues.
Kupper et al., 2024 [33]	Cost and safety outcomes	Complication detection	Digital solution was integrated with hospital EHR and routine clinical practice	Digital solution detected 3 real complications (4.8% of cases) with a sensitivity of 75.0%, specificity of 83.0%, accuracy of 82.0%, positive predictive value of 2.1%, and negative predictive value of 97.0%.
	Patient experience	Adherence to monitoring program	Daily reporting of symptoms for 15 days postoperatively	Complete adherence to the monitoring program was 83.7%.
	Patient experience	Patient satisfaction	NPS	Average NPS score was 81.0, with 95.0% of participants reporting the platform was simple and easy to use.
	Patient experience	Patient perceptions	Qualitative statements	90.5% of patients agreed that they felt safe about being remotely monitored. More than 80.0% of participants said the program contributed to better communication, coordinated care, and patient engagement.
Meinert et al., 2024 [34]	Cost and safety outcomes	Chatbot accuracy and agreement with clinicians	Agreement between chatbot and supervising clinician on 5 key symptoms and overall care management decisions	Average accuracy across 5 key symptoms was 98.7% (SN = 90.7, SP = 99.5); overall outcome decision accuracy was 88.7% (SN = 93.8, SP = 86.3%). Moderate-strong agreement with clinicians in all parameters (kappa = 0.76–0.97).
	Cost and safety outcomes	Chatbot accuracy	Performance metrics	Out of 948 symptom assessments, Dora R1 had 12 (1.3%) errors and 7 (0.7%) false negatives.
		Feasibility	Calls completed autonomously and average call length	96.5% of calls were completed autonomously with a mean call length of 7 min 25 seconds.
	Patient experience	Chatbot acceptability and patient experiences	Semi-structured interviews	Thematic analysis revealed that the chatbot was perceived as an acceptable tool with benefits for patients who had not experienced any complications with their surgery. Participants felt chatbot could benefit both patients and clinicians by increasing convenience, saving time and costs, and providing reassurances. There were some concerns about lack of “human element,” especially during complications. Almost all interviewees found chatbot easy to use and they could understand everything it was saying.
	Patient experience	Chatbot acceptability	NPS	Overall NPS average score was 51.1.
	Patient experience	Chatbot usability	SUS, TUQ	Mean SUS score of 77.8 ± 17.6 and TUQ score of 3.8 ± 0.9.
	Cost and safety outcomes	Cost analysis	Direct cost analysis of face-to-face follow-up vs chatbot	Average staff cost saving off £35.2 per patient compared to standard care.
Rainey et al., 2023 [35]	Patient experience	Patient engagement	Number of patient-generated text messages sent to chatbot	There was no significant difference in message responses of LEP patients compared to EPL patients (12.3 ± 16.6 vs 12.2 ± 12.1, p = 0.96).
	Clinical outcomes	ED visits	Post-op follow-up calls and chart review	LEP patients who were enrolled in the chatbot had a reduction in ED visits, compared to LEP patients that were not enrolled in the chatbot (0.9% vs 8.0%, p = 0.09).
	Clinical outcomes	Readmission	Post-op follow-up calls and chart review	LEP patients who were enrolled in the chatbot had fewer readmissions than control LEP patients (0.0% vs 8.3%, p = 0.01).
	Clinical outcomes	Reoperation	Post-op follow-up calls and chart review	There was no difference in reoperation rates between the chatbot cohort and those not enrolled in the chatbot (0.0% vs 1.5%, p = 1.00).
Rainey et al., 2024 [36]	Patient experience	Patient engagement	Number of text messages sent to chatbot	Patients with insurance other than Medicaid (p < 0.031), females (p < 0.0001), and those undergoing TKA (vs THA) (p > 0.0001) were significantly more likely to engage in chatbot use when looking at total text messages sent as well as messages sent after surgery (p < 0.05). Increasing age significantly correlated with increased total perioperative use (p = 0.005).
	Patient experience	Readmission	Post-op follow-up calls and chart review	Readmitted patients were significantly less likely to engage with the chatbot (3.9 total messages vs 12.7 total messages, p < 0.0001).
	Patient experience	Reoperation	Post-op follow-up calls and chart review	There was no significant difference in chatbot engagement of patients who required reoperation (p = 0.35).
	Patient experience	ED visit	Post-op follow-up calls and chart review	There was no significant difference in chatbot engagement of patients who had an ED visit within 90 days of surgery (p = 0.62).
	Patient experience	Physical function, pain	PRO (Specific scale NA)	Patients with higher pre-op physical function (p = 0.0009) and lower pre-op pain scores (p = 0.038) had significantly higher chatbot engagement. Post-op physical function was associated with increased chatbot engagement (p = 0.04).
	Patient experience	Chatbot queries	Queries were recorded and categorized by StreamMD	Post-op patients seen in the ED most inquired about ambulation (p = 0.005) and weaning from narcotics (p = 0.28). For readmitted patients, discussions around ambulation (p = 0.02) and weaning from narcotics (p = 0.02) were significant. Querying about exercise was associated with patients not being readmitted (p = 0.0009) and not having ED visits postoperatively (p = 0.0001). Patients with a mental health diagnosis (MDD, GAD) were more likely to raise high-acuity topics (p = 0.005).
Yahagi et al., 2024 [37]	Clinical outcomes	Preoperative anxiety	Japanese STAI	IG showed significant reduction in preoperative anxiety that was sustained postoperatively (significant interaction between group and time on STAI scores, p = 0.015).
	Patient experience	Patient satisfaction	4-point scale	Average score for patient satisfaction was 3.0 (IQR: 2–4).
	Patient experience	Improvement in patient understanding of treatment process	4-point scale	Average score for improved patient understanding of treatment process was 2.0 (IQR: 1–2).
	Patient experience	Patient perception of chatbot responses as more relevant than nurses’	4-point scale	Average score of chatbot relevancy compared to anesthesia nurse was 2.0 (IQR: 1–2).

CG: Control Group; ED: Emergency Department; EPL: English as Primary Language; GAD: Generalized Anxiety Disorder; IG: Intervention Group; IQR: Interquartile Range; LEP: Limited English Proficiency; MDD: Major Depressive Disorder; NA: Not Applicable; NPS: Net Promoter Score; NPV: Net Predictive Value; PROMIS: Patient-Reported Outcome Measures Information System; PRO: Patient-Reported Outcome; STAI: State-Trait Anxiety Inventory; SUS: System Usability Scale; TUQ: Telehealth Usability Questionnaire.
