# Supplementary material for: Artificial Intelligence Chatbots in Surgical Care: A Systematic Review of Clinical Applications
Source: J Artif Intell Med Sci. Author manuscript; Available in PMC 2026 Jul 11. (PMC13354317; doi:10.55578/joaims.250603.001)
Supplement: 1 [file NIHMS2118864-supplement-1.pdf]

SUPPLEMENTARY MATERIALS

Search Strategies:

PubMed (MEDLINE)
((chatbot[Title/Abstract] OR "conversational agent"[Title/Abstract]) AND (surgery[Title/Abstract] OR perioperative[Title/Abstract] OR "surgical patients"[Title/Abstract]) AND (intervention[Title/Abstract] OR implementation[Title/Abstract] OR applied[Title/Abstract] OR outcome[Title/Abstract]))

EMBASE
(chatbot:ti,ab OR "conversational agent":ti,ab) AND
(surgery:ti,ab OR perioperative:ti,ab OR "surgical patients":ti,ab) AND
(intervention:ti,ab OR implementation:ti,ab OR applied:ti,ab OR outcome:ti,ab)

Cochrane
(chatbot OR "conversational agent") AND
(surgery OR perioperative OR "surgical patients") AND
(intervention OR implementation OR applied OR outcome)

Google Scholar
"chatbot" AND ("surgical patients" OR "perioperative care") AND ("implementation" OR "outcomes") AND "intervention"

Supplementary Figure 1 | Search strategies for systematic review.

Calculation of time reduction and cost estimates in Meinert et al. [34]

Standard Care:
Total average cost per patient = **£46.96**
Total cost for 100 patients: 100 patients x £46.96 = £4696.00
Average hourly rate for staff (F2F) = 83 + 120 + 52 + 122 + 52 + 626 = £81.83 / hr

Total hours spent in standard care for 100 patients = £4696£81.83 = **57.38 hrs**

Dora R1 Pathway:
Total average cost per patient is **£11.78**
For 100 patients: 100 x £11.78 = £1178.00
Average staff hourly rate (F2F): 83 + 120 + 52 + 122 + 525 = 85.5 / hr
Total call time for 100 patients: (100 patients x 10 min) / 60 minutes = 16.7 hours
Total cost for telephone follow-up (per patient) = £568.33 / 55 patients = £10.33 per patient
Total cost for telephone follow-up (for 100 patients) = £10.33 x 100 patients = £1033.33
Total cost of 100 patients using Dora R1 - total cost of telephone follow-up for 100 patients = £1178-£1033.33 = £145.16 (F2F cost)
Total cost of F2F for 100 patientsaverage staff hourly rate (F2F) = £145.16£85.5 = 1.7 hrs (F2F) per 100 patients

Total time for Dora R1 for 100 patients = 1.7 hrs (F2F) + 16.7 hrs (telephone follow-ups) = **18.4 hrs**

Time saved from using Dora R1 for 100 patients = 57.4 hrs - 18.4 hrs = **39.0 hours**

Supplementary Figure 2 | Calculation of time reduction and cost estimates. This figure presents the step-by-step calculations used to estimate the total time reduction per 100 patients in the chatbot described in Meinert et al. [34].

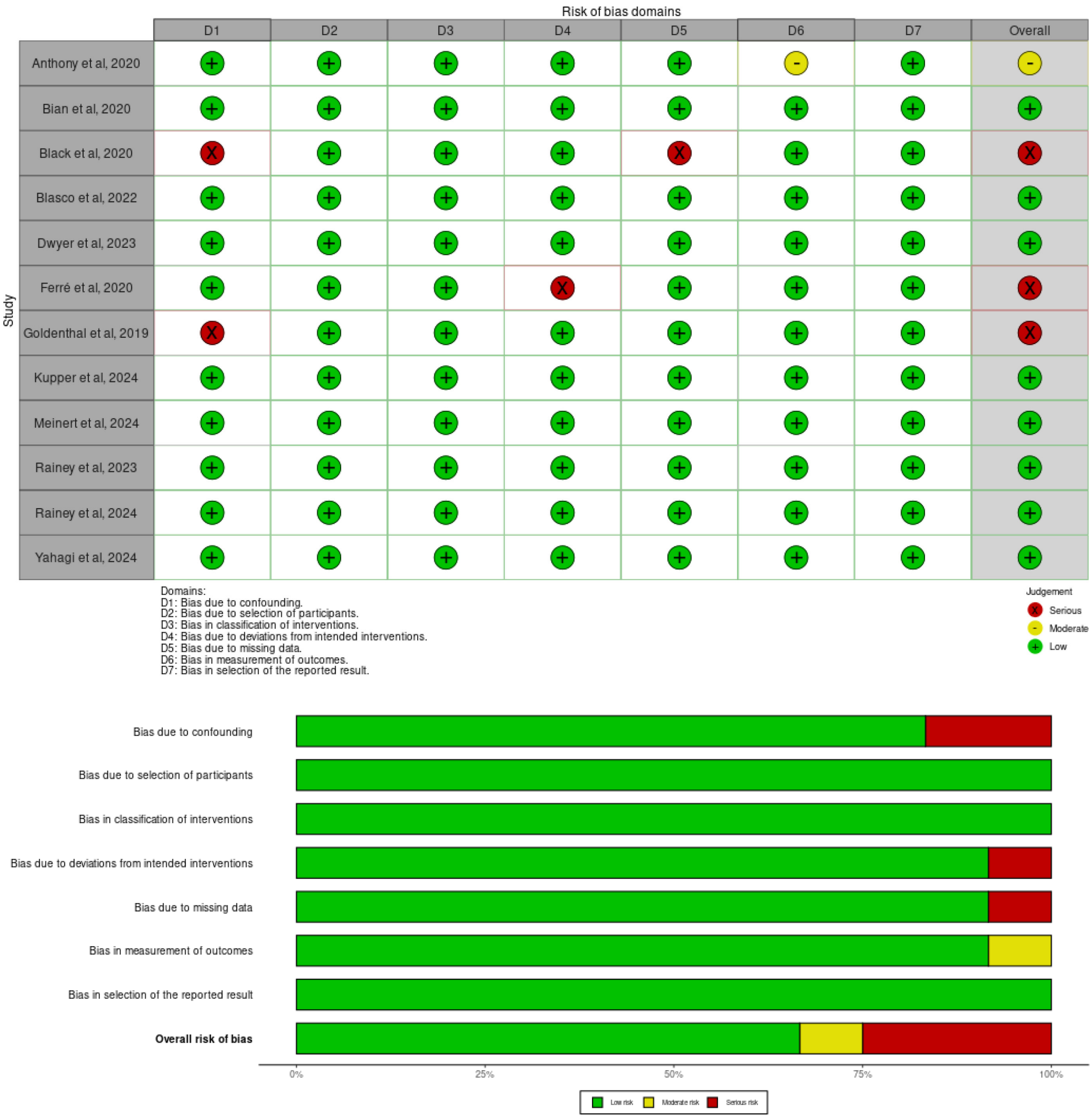

Supplementary Figure 3 | Traffic light plot of risk bias in included studies using ROBINS-I tool (Risk of Bias in Non-randomized Studies-of Interventions).
